# A Pleasant Time

**Published:** 1871-07

**Authors:** 


### A PLEASANT TIME.

The Editors had a pleasant time in Macon on the 5th of July. They met many old friends. Some of the best men of the State were present in the interest of peace and harmony. To Drs. Holt and Hall we tender our special thanks for the kind and delightful entertainments given us and a number of our noble professional brethren present at the Convention. Such re-unions are pleasant, and mark an era in the life of the physician. For whole-souled-cleverness and professional tone, our Macon friends are par-excellence. Of course, such Doctors must have good wives, and our observation in this respect justifies us in thinking that they are not only professional gentlemen, but what is still better, happy husbands.

